# Targeting the Nrf2/ARE Signalling Pathway to Mitigate Isoproterenol-Induced Cardiac Hypertrophy: Plausible Role of Hesperetin in Redox Homeostasis

**DOI:** 10.1155/2020/9568278

**Published:** 2020-09-01

**Authors:** Prema Velusamy, Thangarajeswari Mohan, Divya Bhavani Ravi, S. N. Kishore Kumar, Ashokkumar Srinivasan, Lakshmi Narasimhan Chakrapani, Abhilasha Singh, Saradhadevi Varadharaj, Periandavan Kalaiselvi

**Affiliations:** ^1^Department of Medical Biochemistry, University of Madras, Taramani, Chennai 600113, India; ^2^Cardiovascular Medicine, The Ohio State University, Columbus, Ohio 43210, USA

## Abstract

Cardiac hypertrophy is the underlying cause of heart failure and is characterized by excessive oxidative stress leading to collagen deposition. Therefore, understanding the signalling mechanisms involved in excessive extracellular matrix deposition is necessary to prevent cardiac remodelling and heart failure. In this study, we hypothesized that hesperetin, a flavanone that elicits the activation of Nrf2 signalling and thereby suppresses oxidative stress, mediated pathological cardiac hypertrophy progression. A cardiac hypertrophy model was established with subcutaneous injection of isoproterenol in male Wistar rats. Oxidative stress markers, antioxidant defense status, and its upstream signalling molecules were evaluated to discover the impacts of hesperetin in ameliorating cardiac hypertrophy. Our results implicate that hesperetin pretreatment resulted in the mitigation of oxidative stress by upregulating antioxidant capacity of the heart. This curative effect might be owing to the activation of the master regulator of antioxidant defense system, known as Nrf2. Further, analysis of Nrf2 revealed that hesperetin enhances its nuclear translocation as well as the expression of its downstream targets (GCLC, NQO1, and HO-1) to boost the antioxidative status of the cells. To support this notion, *in vitro* studies were carried out in isoproterenol-treated H9c2 cells. Immunocytochemical analysis showed augmented nuclear localization of Nrf2 implicating the action of hesperetin at the molecular level to maintain the cellular redox homeostasis. Thus, it is conceivable that hesperetin could be a potential therapeutic candidate that enhances Nrf2 signalling and thereby ameliorates pathological cardiac remodelling.

## 1. Introduction

Cardiac hypertrophy is the heart's response to multiple forms of stress with an adaptive increase in cardiac mass. At the molecular level, cardiac hypertrophy is characterized by an increase in myocardial cell size, a higher degree of sarcomeric organization, reactivation of the fetal gene program, and changes in gene transcription and translation resulting in enhanced protein synthesis [1–3]. Although pathological hypertrophy is initially beneficial, prolonged hypertrophy leads to heart failure and sudden death [4, 5]. Pathological conditions such as hypertension and myocardial infarction can lead to hypertrophy [6]. Catecholamines, including phenylephrine (PE) and isoproterenol (ISO), play key roles in inducing cardiac hypertrophy [7–9].

Oxidative stress has been identified as one of the key contributing factors in the progression and development of cardiac hypertrophy [10]. Reactive oxygen species (ROS) can activate a wide variety of hypertrophy signalling kinases and transcription factors [11], and excessive ROS can lead to pathological remodelling, MMP activation, fibrosis, apoptosis, and contractile dysfunction [12]. ROS have been shown to stimulate cardiac fibroblast proliferation [13] and MMP activation [14] leading to fibrosis and matrix remodelling.

The induction of proteins that increase the antioxidant capacity of cells and enable restoration of intracellular redox homeostasis is a major protective mechanism against the damaging consequences of oxidative stress. The expression of many antioxidant and cytoprotective proteins, including classic phase 2 detoxification enzymes, chaperone proteins, antioxidant enzymes, and proteins in the proteasomal degradation pathway, is regulated by Nrf2 [15, 16] that belongs to the Cap “n” Collar (CNC) family of basic leucine zipper (bZip) transcription factors that regulate the ARE- (antioxidant response element) mediated basal and inducible gene expression. Keap1 is a cytoplasmic protein that is associated with Nrf2 [17] and controls Nrf2 at the posttranslational level by cytoplasmic retention and rapid degradation through the proteasome system [18].

The transcription factor, Nrf2 (nuclear factor erythroid-derived 2-like 2), is a critical regulator for maintaining structural and functional integrity of the heart under conditions of abnormal stress [19], and also, it is an important component in antioxidant defense in cardiovascular diseases such as atherosclerosis, hypertension, and heart failure [19, 20]. It is increasingly apparent that Nrf2 is important for vascular integrity and long-term endothelial function [21]. In the nucleus, Nrf2 combines with a small protein called Maf to form a heterodimer and binds to the antioxidant response element in the upstream promoter region. This initiates the transcription of a number of antioxidative genes, namely, heme oxygenase-1 (HO-1), NAD(P)H dehydrogenase (quinone 1) (NQO1), superoxide dismutases (SODs), catalase (CAT), glutathione-S-transferase (GST), Glutamate-Cysteine Ligase Catalytic Subunit (GCLC), and glutathione peroxidase (GPx) [22].

Therefore, the activation of Nrf2 provides a novel mechanism to protect the heart against pathological changes by maintaining systemic redox balance [23–25]. Hesperetin a citrus bioflavonoid has been reported for its cardioprotective effect [26, 27]. Hesperetin is shown to possess antihypertensive, hypolipidemic, anti-inflammatory, and antioxidant activity along with a potential to boost Nrf2. These features deem it to be an ideal candidate to treat hypertrophy. However, there is no ample scientific evidence to prove its efficacy and mechanism of action in the oxidative stress-mediated pathological conditions like cardiac hypertrophy. Hence, the present study was designed to elucidate the protective role of hesperetin in preventing isoproterenol-induced cardiac hypertrophy and to delineate the signalling pathway responsible for this cardioprotective effect.

## 2. Methods

### 2.1. Animals and Treatment

Male albino rats of Wistar strain were used for this study. Animals were obtained from the Central Animal Facility, Taramani Campus, University of Madras, and maintained as per national guidelines and protocols, approved by the Institutional Animal Ethics Committee (IAEC Number: 01/23/2012). All experiments with animals were performed in compliance with the relevant laws and institutional guidelines. The animals were housed under conditions of controlled temperature (25 ± 2°C) with 12/12 h light/dark cycle and were given food and water *ad libitum*. Cardiac hypertrophy was induced by subcutaneous injections of isoproterenol (5 mg/kg body weight) for seven days. Animals were divided into four groups: (1) control rats (received vehicle alone); (2) hypertrophy-induced rats (received isoproterenol hydrochloride 5 mg/kg body weight, subcutaneously for 7 days); (3) rats pretreated with hesperetin (received hesperetin suspended in 0.5% methyl cellulose orally for 30 days) and given isoproterenol 5 mg/kg body weight, subcutaneously for 7 days; and (4) rats pretreated with hesperetin (received hesperetin suspended in 0.5% methyl cellulose orally for 30 days).

At the end of the experimental period, rats were sacrificed under ketamine anaesthesia (22 mg/kg bw/ip). Hearts were excised, washed in ice-cold saline, blotted dry, and weighed, and part of the heart was homogenized in ice-cold tissue lysis buffer. A portion of the heart was used to isolate RNA. A 10% heart homogenate was prepared using 0.01 M Tris-HCl buffer (pH 7.4) from a portion of the tissue, and the remaining part of the tissue was stored at –80°C until further analysis. A detailed list of methods used in the study has been given as a separate supplementary material (available here).

### 2.2. Cell Culture

Rat cardiomyoblast cells (H9c2) were grown on tissue culture flasks in Dulbecco's modified Eagle's complete cell culture medium (DMEM, Invitrogen), supplemented with 10% fetal bovine serum, 1% penicillin, and streptomycin, and maintained in 95% air and 5% CO_2_ at 37°C. For protein extraction, cells were seeded in 10 cm dishes, starved for 24 hrs in serum-free DMEM, and used for experiments. Cells were treated with hesperetin at a concentration of 10 *μ*M for 12 hours followed by isoproterenol at a concentration of 10 *μ*M for 24 hours.

### 2.3. Statistical Analysis

The results are expressed as the mean ± Standard Error of Mean (SEM). Differences between groups were analysed by one-way analysis of variance (ANOVA) using the SigmaPlot Software (Version: 11.0). Post hoc testing was performed for intergroup comparisons using the Student-Newman-Keuls and Tukey post hoc tests; significance (^∗∗∗^*p* < 0.001, ^∗∗^*p* < 0.01, and ^∗^*p* < 0.05) has been given by respective symbols in figures.

## 3. Results

### 3.1. Index of Cardiac Hypertrophy

**Figure 1:**
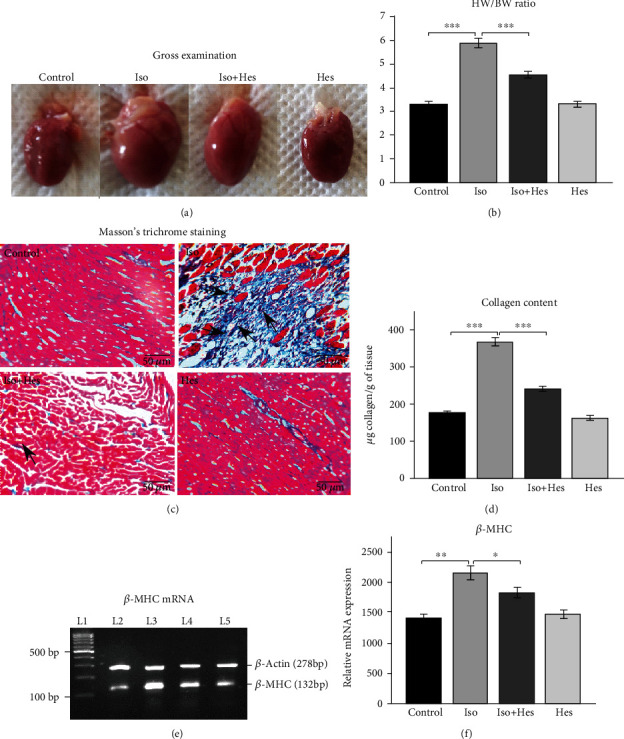
Hesperetin reduces cardiac hypertrophy in rats. (a) Representative images of gross examination of the heart; (b) heart weight to body weight ratio; (c) representative images of Masson trichrome staining, scale bars: 50 *μ*m; heart light-field photomicrographs of Masson's trichrome-stained sections (40x). Fibrosis is indicated by black arrow marks: (i) control heart shows normal architecture, (ii) isoproterenol-administered rats show moderate to severe fibrosis, (iii) isoproterenol+hesperetin-treated rats show mild fibrosis, and (iv) hesperetin alone-treated rats show no fibrosis. (d) Quantification of fibrosis measured by collagen (hydroxyproline) assay. (e, f) *β*-MHC mRNA expression levels: L1: marker, L2: control, L3: isoproterenol, L4: isoproterenol+hesperetin, and L5: hesperetin. Data are expressed as the mean ± SEM of the band intensity, and each experiment conducted three repeats per condition. *n* = 6 for each group. Statistical significance (^∗∗∗^*p* < 0.001, ^∗∗^*p* < 0.01, and ^∗^*p* < 0.05) was calculated by Student-Newman-Keuls and Tukey post hoc tests. Iso: isoproterenol-administered rats; Iso+Hes: isoproterenol-administered rats pretreated with hesperetin.

The heart weight to body weight ratio (HW/BW) was used as an index of cardiac hypertrophy. The size of the heart (Figure 1(a)) and the HW/BW ratio (Figure 1(b)) were found to be higher in the isoproterenol-administered rats when compared to control rats. Upon pretreatment with hesperetin to the isoproterenol-administered rats, there was a marked decrease in the heart size and HW/BW ratio. To evaluate the degree of collagen deposition in the heart tissue, Masson's trichrome staining was carried out. Histological staining showed moderate to severe fibrosis in isoproterenol-induced hypertrophied rat hearts, while minimum fibrosis was observed in the hesperetin-pretreated groups (Figure 1(c)). Besides, when measuring the collagen content (as a measure of cardiac fibrosis), it showed elevated levels of collagen in the heart of isoproterenol-administrated animals when compared to those of the control groups, whereas the collagen content was reduced significantly in the rats pretreated with hesperetin (Figure 1(d)). Since hypertrophy-associated expression of fetal gene *β*-MHC is the widely used marker of the condition, we analysed the gene expression of *β*-MHC (Figures 1(e) and 1(f)). The levels of *β*-MHC was found to be increased in the hypertrophic rat heart. This increase in mRNA expression of *β*-MHC in isoproterenol-administered rats was found to be refrained by hesperetin. The cell size as evident from the Alexa Fluor 488 phalloidin staining (Supplementary Figure 1) was found to be increased significantly in the isoproterenol-treated H9c2 cells when compared to the control. This increase in cell size was not found in the hesperetin-treated cells.

### 3.2. Oxidative Stress Markers

**Figure 2:**
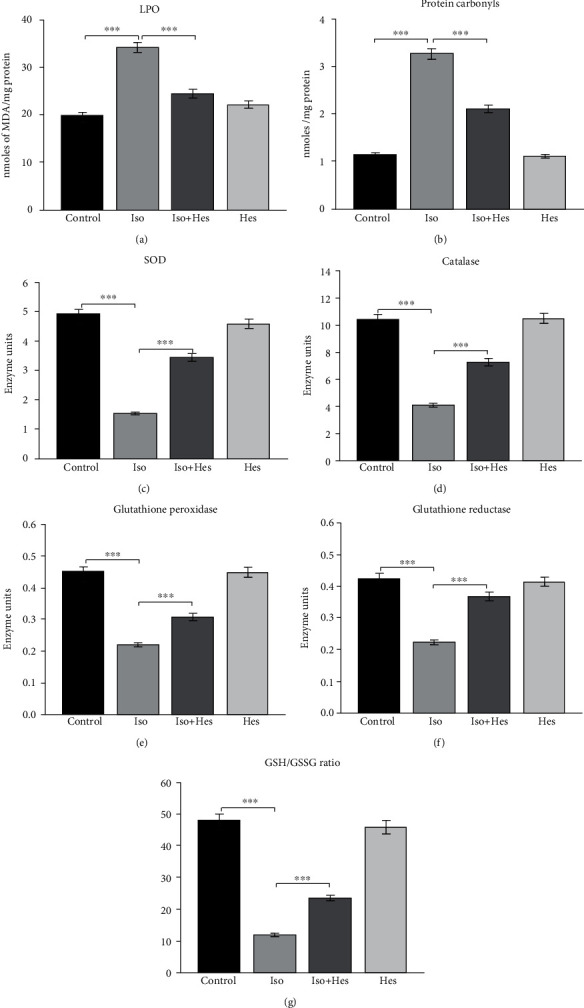
Analysis of oxidative stress markers that were modified in cardiac hypertrophy. (a) Comparison of lipid peroxidation. Units: LPO (nmoles of MDA/mg protein). (b) Protein carbonyl content between control and experimental groups; PC (nmole/mg protein). (c) Hesperetin augments the antioxidant status. Units: superoxide dismutase—amount of enzyme required to prevent 50% autooxidation of pyrogallol/min/mg protein. (d) Catalase (*μ*moles of H_2_O_2_ consumed/min/mg protein). (e) Glutathione peroxidase (*μ*moles of GSH oxidized/min/mg protein). (f) Glutathione reductase (*μ*moles of NADPH consumed/min/mg protein). (g) GSH/GSSG ratio. Data are expressed as the mean ± SEM, and each experiment conducted three repeats per condition. *n* = 6 for each group. Statistical significance (^∗∗∗^*p* < 0.001) was calculated by Student-Newman-Keuls and Tukey post hoc tests.

Oxidative damage to lipids was assessed by measuring the lipid peroxidation.  Figure 2(a) shows the MDA levels in the cardiac tissue of the experimental groups. MDA levels reflect the levels of lipid peroxidation. The levels of MDA were increased to a greater extent in myocardial tissues of hypertrophied rat hearts when compared to those of the control rats. Hesperetin-treated rats showed a drop in the MDA levels when compared to the isoproterenol group. Proteins are major targets of ROS and secondary by-products of lipid oxidation, and oxidatively modified proteins are routinely analysed to indicate excessive oxidative stress [28].  Figure 2(b) shows the level of protein carbonyls in cardiac tissue of all the experimental groups. Carbonyl levels were found to be increased in the hypertrophied rat heart when compared to control rats, while hesperetin-treated rats showed a marked decline in the protein carbonyl levels when compared to the isoproterenol-administered rats. From the above results, it can be inferred that hesperetin prevents oxidative damage to the lipids and proteins.

### 3.3. Antioxidant Capacity

Stimulation of *β*1-adrenergic receptors rapidly generates ROS as well as impairs the total cellular antioxidant capacity. The activity of the enzymatic antioxidants SOD, CAT, GPx, and GR was found to be declined in the isoproterenol-administered rats when compared to the control rats (Figures 2(c)–2(f)). However, these activities were found to be significantly higher in the hesperetin-pretreated groups implying the antioxidant potential of hesperetin. Having said that, the ratio of GSH/GSSG (Figure 2(g)) plays a major role in regulating the redox status during hypertrophy, and inevitably, it is lowered in the isoproterenol-treated animals, while hesperetin treatment augmented the GSH/GSSG ratio in the cardiac hypertrophic rats. Therefore, it is apparent that being an antioxidant, hesperetin is capable of boosting the endogenous antioxidant defense system by enhancing the antioxidant enzyme activity and regulating the GSH/GSSG ratio.

### 3.4. Expression and Activity of ARE Induced Cytoprotective Enzymes

**Figure 3:**
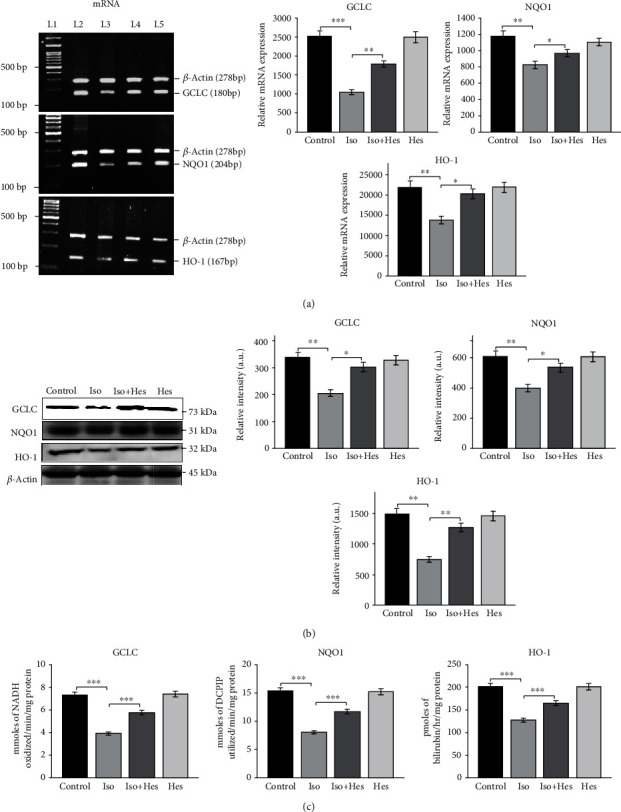
mRNA levels, protein expression, and activity of GCLC, NQO1, and HO-1. (a) Gene expression levels of GCLC, NQO1, and HO-1 (left panel); L1: marker; L2: control; L3: isoproterenol; L4: isoproterenol+hesperetin; L5: hesperetin. Relative mRNA levels—band intensity normalized with *β*-actin (right panel). (b) Immunoblot analysis of antioxidant protein expression in the heart. Actin served as a loading control. The protein levels were normalized to the actin level. (c) Bar plots showing the activity of GCLC, NQO1, and HO-1. Results are shown as the mean ± SEM of three separate experiments. Statistical significance (^∗∗∗^*p* < 0.001, ^∗∗^*p* < 0.01, and ^∗^*p* < 0.05) was calculated by Student-Newman-Keuls and Tukey post hoc tests.

Since we observed an increase in the antioxidant, we investigated the gene expression, protein levels, and the activity of cytoprotective enzymes GCLC, HO-1, and NQO1 (Figures 3(a)–3(c)). Isoproterenol-induced hypertrophic rats showed a significant decline in the expression and activity of GCLC, HO-1, and NQO1. The gene expression, protein levels, and the activity of GCLC, HO-1, and NQO1 were significantly increased in rat hearts treated with hesperetin when compared to the isoproterenol-administered rats.

### 3.5. Effect of Hesperetin on mRNA and Protein Expression of Nrf2

**Figure 4:**
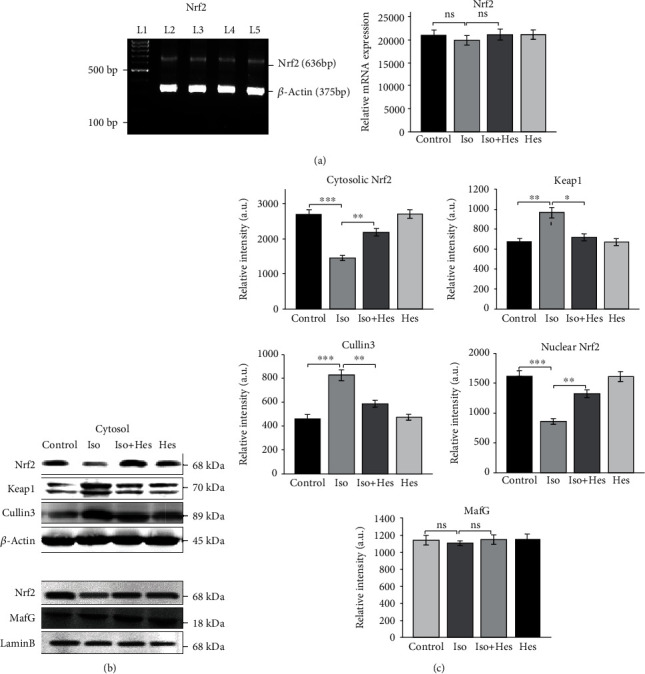
Hesperetin mediated activation of Nrf2. (a) RT-PCR analysis of Nrf2 (left panel) and the relative mRNA levels (right). (b) Immunoblot analysis of cytosolic and nuclear Nrf2 and its regulators. (c) Bar plots showing the densitometric analysis of cytosolic and nuclear Nrf2, Keap1, Cullin3, and MafG. Actin served as a loading control. The band intensity was normalized to that of actin and Lamin B. Results are shown as the mean ± SEM of three separate experiments. Statistical significance (^∗∗∗^*p* < 0.001, ^∗∗^*p* < 0.01, and ^∗^*p* < 0.05; ns: not significant) was calculated by Student-Newman-Keuls and Tukey post hoc tests.

As the above results implicated an overall enhancement in the transcription of all the antioxidant genes, we hypothesized that the reason behind the increased expression of antioxidant enzymes and their activities in the hesperetin-treated rats might be due the upregulation of its upstream molecule Nrf2 during cardiac hypertrophy. Therefore, the mRNA and protein expression of Nrf2 and its regulators were analysed using reverse transcriptase PCR and western blotting. The mRNA expression of Nrf2 (Figure 4(a)) was found to be unaltered in all the experimental groups, while the protein expression (both nuclear and cytosolic) was considerably reduced in isoproterenol-induced hypertrophied rat hearts when compared to that of the control rat hearts (Figure 4(b)). Hesperetin was found to increase the protein levels of Nrf2 in the isoproterenol-induced hypertrophic rat hearts. In the nucleus, Nrf2 heterodimerizes with MafG (a transcriptional coactivator) to elicit its response against oxidative stress. The expression of MafG was found to be unaltered between the experimental groups.

### 3.6. Expression of Negative Regulators of Nrf2

As indicated above, the gene expression of Nrf2 was unaltered while the protein levels were reduced significantly in the hypertrophic hearts. We hypothesized that the possible reason for this disparity might be an increase in the degradation of Nrf2 at a posttranslational level. To address this hypothesis, we investigated whether the expression of Keap1 and Cullin3 (Figure 4(b)), the proteins involved in the regulation of Nrf2, was altered. Interestingly, western blot analysis revealed an increased expression of both Keap1 and Cullin3, the proteins involved in the ubiquitination and degradation of Nrf2. Hesperetin was found to decrease the expression of Keap1 and Cullin3.

### 3.7. Effect of Hesperetin on the Superoxide Levels in H9c2 Cells

**Figure 5:**
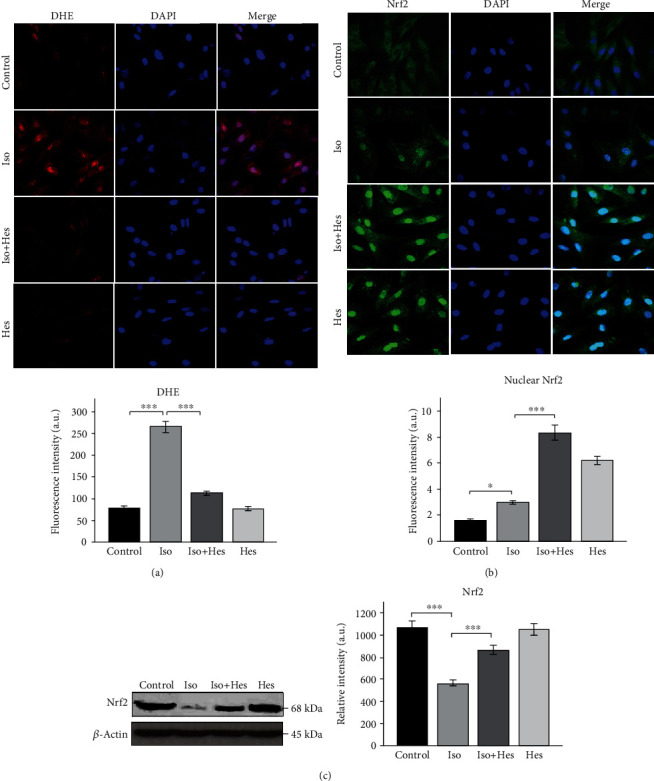
(a) Hesperetin suppressed intracellular ROS production. Representative images of DHE staining in H9c2 cells. Nuclei were counterstained with DAPI. Bar plots showing the fluorescence intensity (AU). (b) Nrf2 nuclear translocation in H9c2 cardiomyoblasts. Representative images of immunohistochemical staining for the levels of Nrf2. Histograms represent the quantification (nuclear) of fluorescence intensity. (c) Immunoblot analysis of Nrf2. Immunoblot analysis of Nrf2 levels in H9c2 cells. Actin was used as a loading control. Quantification of the protein levels normalized to actin is shown. Results are shown as the mean ± SEM of three separate experiments. Statistical significance (^∗∗∗^*p* < 0.001, ^∗∗^*p* < 0.01, and ^∗^*p* < 0.05) was calculated by Student-Newman-Keuls and Tukey post hoc tests.

To study the antioxidant potential of hesperetin, we checked the level of oxidative stress in the isoproterenol-treated H9c2 cells with or without hesperetin treatment. The superoxide generation (Figure 5(a)) as evident from the cytochemistry results showed a significant increase in isoproterenol-treated cells when compared to that of the control cells. Also, there was a marked reduction of superoxide radical generation in hesperetin-pretreated cells when compared to that of isoproterenol alone-treated cells.

### 3.8. Expression and Nuclear Translocation of Nrf2 in H9c2 Cells

Immunocytochemistry results showed a slight increase in the nuclear translocation of Nrf2 when compared to the untreated control cells (Figure 5(b)), whereas western blot analysis of the whole cell extract revealed a significant decrease in the total Nrf2 protein expression (Figure 5(c)) in the isoproterenol-treated cells when compared to the control untreated cells. Hesperetin-pretreated cells showed a significant increase in both the protein levels and the nuclear translocation of Nrf2 as measured by the fluorescence intensity when compared to isoproterenol alone-treated cells.

## 4. Discussion

Oxidative stress is a major culprit for the cause of cardiac hypertrophy and heart failure. Our results highlighted the impact of hesperetin as a novel therapeutic potential agent by targeting Nrf2 against isoproterenol-induced cardiac hypertrophy. The heart weight to body weight ratio is an established measure of cardiac hypertrophy [29], and the induction of fetal gene expression (*β*-MHC) in the pathologically hypertrophied myocardium [30] results in myocardial dysfunction. The observed increased measure of these two in the present study correlates with the development of cardiac hypertrophy due to isoproterenol administration. In fact, increased expression of the *β*-MHC gene is observed in the most of the cardiac hypertrophy experimental models, and heart failure as the expression of *β*-MHC in place of *α*-MHC directly affects cardiac function, as judged by a reduction in myofibrillar ATPase activity and reduced shortening velocity of cardiac myofibers in animals [31]. Hesperetin was found to ameliorate the increased expression of fetal genes and regulates hypertrophy negatively as there was a significant decline in the HW/BW ratio and the expression of *β*-MHC.

Cardiac fibrosis which is considered to be a critical event during pathological hypertrophy is a multifactorial process involving complex interactions between stimulatory and inhibitory factors, leading to increased collagen deposition in the myocardium [32]. As a critical step in response to myocardial injury, fibroblasts are activated into *α*-smooth muscle actin positive myofibroblasts which can generate extracellular matrix proteins such as type I collagen resulting in the imbalance between the synthesis and degradation of collagen [33] leading to left ventricular fibrosis. Sustained activation of cardiac *β*-adrenergic receptor (*β*-AR) can lead to the development of cardiac remodelling [34]. Isoproterenol has been shown to increase the expression of TIMP-1 (tissue inhibitor of metalloproteinase) and MMP-1 (matrix metalloproteinase-1) [35, 36]. Isoproterenol has been known to increase cardiac oxidative stress, and the resulting ROS generation triggers MAP kinase activation. Also, under chronic isoproterenol infusion, increased ROS play important roles in extracellular matrix biosynthesis, leading to the alteration in wall stiffness and cardiac function [37]. The degree of fibroblast activation and subsequent collagen deposition is considered to be a significant predictor of heart failure progression in both experimental animal models and in human patients [38]. Increased deposition of collagen proteins has been observed in patients with hypertension, dilated cardiomyopathy, and end-stage heart failure [39]. Consistent with these previous studies, we also observed increased levels of total collagen in the isoproterenol-administered rats. The present observations along with the literature support [40] substantiate that hesperetin can culminate the cardiac events that trigger cardiac fibrosis, and it could act as a potent antihypertrophic agent.

To further confirm hesperetin's antifibrotic activity, sections of the heart were subjected to Masson's trichrome staining which is specific for collagen. The results implicate that hesperetin could reduce the collagen deposition in the isoproterenol-administered rats. Studies by Liu and coworkers [41] reported that hesperetin could block cardiac fibrosis and attenuate the expression of several fibrotic mediators induced by chronic pressure overload. They have demonstrated that hesperetin abrogates TGF*β*1 mRNA expression and Smad2/3 phosphorylation in hypertrophic hearts and thus inhibits fibrosis.

In the cardiovascular system, ROS can activate a broad variety of hypertrophy-promoting kinases and transcription factors, and oxidative stress has been identified as one of the key contributing factors in the development of cardiac hypertrophy [42]. The levels of TBARS and protein carbonyls in the hypertrophied heart demonstrate that the oxidative capacity of a cell is altered, and it has been authenticated as a pivotal factor in the development of cardiac hypertrophy and heart failure [43]. Production of LPO may be due to the oxidation of isoproterenol to semiquinone which reacts with oxygen to produce superoxide anions and H_2_O_2_ [44]. On the other hand, protein oxidation affects signal transduction mechanisms, enzyme activity, heat stability, and proteolysis susceptibility, which leads to pathological changes [45]. The increased production of protein carbonyls might be due to the aminochromes formed by the cyclization of isoproterenol which are highly reactive molecules that can cause oxidation of protein sulfhydryl groups and deamination catalysis [46]. However, hesperetin treatment was found to mitigate the formation of lipid peroxidation and protein carbonyl content to a considerable extent.

Superoxide dismutase, catalase, glutathione peroxidase, and glutathione reductase are closely related antioxidant enzymes that play a vital antioxidant role in human health, conferred by their scavenging of the reactive oxygen species. The reduction in the above-mentioned activity might be due to the increased oxidative stress in the isoproterenol group. However, the activity of the enzymes is restored upon hesperetin treatment. Alteration in the GSH/GSSG ratio is an important factor that mediates the generation of excess free radical generation which is abrogated in the hesperetin supplementation. Herein, being an endogenous antioxidant, reduced glutathione is found at millimolar concentrations in most of the cells [47] and participates in the detoxification process either through enzymatic reduction of a disulfide or by *de novo* synthesis [48].

The enzyme catalyzing the first and rate-limiting step in *de novo* GSH synthesis is GCLC; therefore, its gene regulation is one of the major determinants of GSH homeostasis [49]. Our study indicates that the expression and activity of the enzyme GCLC are significantly augmented with hesperetin treatment to cardiac hypertrophic rats. So far, there are no reports stating a direct action of hesperetin or hesperidin on the expression or activity of GCLC. The increased GCLC activity observed in the hesperetin-pretreated rats might have been the reason for the restoration of GSH homeostasis in these rat hearts as reported in the earlier section. NQO1 represents a family of flavoproteins that catalyze the two-electron reduction of quinones and their derivatives. NQO1 also possess other important biological activities including anti-inflammatory effects, direct scavenging of superoxide anion radicals, and stabilization of p53 and other tumor suppressors [50]. This study is the first of its kind to report the role of hesperetin in enhancing the expression and the activity of NQO1. HO-1 catalyzes the rate-limiting step in the oxidative degradation of cellular heme that liberates iron, carbon monoxide (CO), and biliverdin. In addition to this, HO-1 also plays an important function in various physiological and pathophysiological states associated with cellular stress [51, 52]. Our findings ascertain that hesperetin not only involves itself directly in reducing the oxidative stress but also acts at multifarious levels in increasing the expression of enzymes involving in antioxidant regeneration, which directed us to focus on the upstream regulators of all these antioxidant enzymes, Nrf2.

From our results, we observed an interesting fact that the mRNA expression of Nrf2 is unaltered in the isoproterenol-administered rats while the protein levels as evident from the western blotting data are significantly decreased when compared to the control group. Previous studies have reported that cardiac hypertrophy decreases the protein expression of Nrf2 in mice after four weeks of pressure overload [25]. Although the discrete molecular mechanism behind the decreased Nrf2 protein levels is not known, it is suggested that a decrease in Nrf2 might play a critical role in the regulation of maladaptive cardiac remodelling and the transition of cardiac hypertrophy to heart failure [23] thereby contributing to the pathological cardiac remodelling. Hesperetin-treated rats showed a significant increase in the expression and nuclear translocation of Nrf2 as evident from the present study and previous studies from our laboratory [26]. Also, Nrf2 is known to dimerize with small Maf protein, MafG, to bind to antioxidant response elements (AREs) located in the regulatory regions of cellular defense enzyme genes [22]. Interestingly, we found no alteration in the expression of MafG in any of the experimental groups when compared to the control group. This led us to explore the possible reason for the encountered low levels of Nrf2 despite its normal transcription rate. Therefore, we analysed the expression of the proteins involved in the degradation of Nrf2 (Keap1, Cullin3) which intricately control the steady state levels of Nrf2 post-translationally.

Cullin3 (Cul3) serves as a scaffolding protein that is bound to both Rbx1 and Keap1, which is the substrate adaptor protein that binds to Nrf2. Under normal conditions, Keap1 brings Nrf2 into the Cul3-Rbx1 complex and enables ubiquitin conjugation onto specific lysine residues located within the N-terminal Neh2 domain of Nrf2 [53]. Conversely, following exposure of cells to a wide variety of chemical inducers of Nrf2-dependent transcription, Keap1-dependent ubiquitination of Nrf2 is blocked, enabling Nrf2 to accumulate in the nucleus and activate expression of Nrf2-dependent genes. In the present study, the protein expression of Keap1 and Cullin3 was found to be increased in the hypertrophied rat heart when compared to that of the control rat heart. Keap1-Cullin3-mediated ubiquitination might be the possible reason for the decreased levels of Nrf2 in the isoproterenol-induced rat heart when compared to the control rat heart. The expression of both Keap1 and Cullin3 has been found to decrease upon hesperetin pretreatment.

As the morphological, biochemical, and electrophysiological characterization of H9c2 cells have revealed that this cell line has preserved elements of electrical and hormonal signalling pathways found in adult cardiac cells [54] and they mimic a wide range of hypertrophic behaviour exhibited by primary cardiomyocytes in response to hypertrophic agents [55], an *in vitro* approach using H9c2 cell lines was adopted for evaluation of the efficacy of hesperetin in preventing cardiac hypertrophy.

Isoproterenol treatment has been shown to induce oxidative stress in H9c2 cells, and the superoxide anion generation was found to be higher in isoproterenol-treated cells [54]. Studies by Liang et al. [56] have shown isoproterenol-induced cardiotoxicity to be related to the formation of oxygen-free radicals through a variety of oxidative products. Our results corroborate with the studies done by Kim et al. [57] who had reported that high glucose-induced ROS overproduction *in vitro* was reversed by pretreatment of cells with hesperetin. Correspondingly, the decreased production of ROS is due to the fact that hesperetin may trigger the activation of Nrf2. Therefore, we were keen to explore the expression and nuclear translocation of the transcription factor Nrf2 as it serves as a negative feedback regulator in these pathological conditions.

We noticed a mild increase in the nuclear Nrf2 levels of the isoproterenol-treated cells, when compared to the untreated cells. This might be due to the fact that under normal physiological conditions, Nrf2 is bound to Keap1 in the cytoplasm, directing it to ubiquitination and constitutive degradation [17, 18]. Whereas in the isoproterenol-treated cells, the resulting oxidative stress might have led to the mild elevation in the nuclear Nrf2. However, it is evident from the western blot data that the total Nrf2 is significantly decreased in the isoproterenol group when compared to the control. Li et al. [23] have reported that Nrf2 expression and activity are enhanced during the early stages of cardiac adaptive hypertrophy and decreased in the process of maladaptive responses to sustained hemodynamic stress. Nrf2 levels are increased in response to pathological oxidative stress in the heart; however, during prolonged stress conditions, Nrf2 may be exhausted or downregulated, leading to the failure to maintain the redox homeostasis. Consequently, the persistent oxidative stress induces cardiac remodelling and finally heart failure. However, the Nrf2 protein levels and the nuclear translocation as evident from the immunocytochemistry are significantly increased in hesperetin-pretreated cells. Similar results have been reported in studies using ARPE-19 cells wherein hesperetin was found to significantly increase the total and nuclear levels of Nrf2 in a time-dependent manner [58]. However, the exact mechanism behind this is yet to be studied. Literature evidence states that hesperidin and hesperetin are found to augment the cellular antioxidant defense via the Nrf2 pathway [59]. Therefore, it is conceivable that Nrf2 signalling is critical for suppressing these oxidative stress responses which leads to pathological remodelling of the heart.

## 5. Conclusion

To conclude, increased oxidative stress is one of the major causes of cardiac hypertrophy and heart failure. Our results highlight a novel therapeutic potential of hesperetin by targeting Nrf2 against isoproterenol-induced cardiac hypertrophy. Hesperetin was found to be a potent antioxidant that prevents isoproterenol-induced cardiac hypertrophy. This is one of the first studies to suggest that hesperetin can act as a negative modulator of cardiomyocyte hypertrophy via the activation of Nrf2. However, further studies are required to examine the transcriptomic/metabolomic changes in Nrf2 during cardiac hypertrophy.

## Data Availability

The authors confirm that the data supporting the findings of the study are provided within the manuscript.

## Abbreviations

Nrf2: Nuclear factor erythroid-derived 2-like 2
Keap1: Kelch-like ECH-associated protein 1
HO-1: Heme oxygenase-1
NQO1: NAD(P)H quinone dehydrogenase 1
SOD: Superoxide dismutases
GCLC: Glutamate-Cysteine Ligase Catalytic Subunit
GPx: Glutathione peroxidase
GR: Glutathione reductase
Iso: Isoproterenol
Hes: Hesperetin.

## Supplementary Materials

Supplementary Materials Supplementary Figure 1: cell size measurement. Representative images of Alexa Fluor 488 phalloidin staining in H9c2 cells. Nuclei were counterstained with DAPI. Bar plots showing the relative cell size measured using ImageJ software. Results are shown as the mean ± SEM of three separate experiments. Statistical significance (^∗∗∗^*p* < 0.001) was calculated by Student-Newman-Keuls and Tukey post hoc tests.
