# Supplementary material for: Targeting the Nrf2/ARE Signalling Pathway to Mitigate Isoproterenol-Induced Cardiac Hypertrophy: Plausible Role of Hesperetin in Redox Homeostasis
Source: Oxid Med Cell Longev. 2020 Sep 1;2020:9568278. doi: 10.1155/2020/9568278 (PMC7482027; doi:10.1155/2020/9568278)
Supplement: Supplementary Materials — Supplementary Figure 1: cell size measurement. Representative images of Alexa Fluor 488 phalloidin staining in H9c2 cells. Nuclei were counterstained with DAPI. Bar plots showing the relative cell size measured using ImageJ software. Results are shown as the mean ± SEM of three separate experiments. Statistical significance (∗∗∗p < 0.001) was calculated by Student-Newman-Keuls and Tukey post hoc tests. [file 9568278.f1.zip › supplementary material_omcl_3122523)08172020.docx]

**Supplementary material**

**Methods**

**Reagents**

Hesperetin and Isoproterenol hydrochloride were purchased from Sigma chemicals, St. Louis, MO, USA. Antibody against Nrf2 was purchased from Abcam, USA, antibodies against Keap1 and Cullin3 were from Thermo scientific, HO-1, NQO1and GCLC antibodies were purchased from Santa Cruz Biotechnology Inc, USA and β-actin antibody was purchased from Cell Signaling Technology, USA. TRIzol reagent was from Life Technologies, USA. All other chemicals and reagents used were of analytical grade.

**Masson’s Trichrome Staining**

To determine the extent of fibrosis in the heart, paraffin-embedded sections were stained with Masson’s trichrome stain using a modified method of Kiernan (2008) and Jin et al. (2017). Briefly, hearts were excised from anaesthetized rats, washed in PBS, fixed overnight in 4% paraformaldehyde, and embedded in paraffin. After serial sectioning of hearts (apex to base) 9-15, 5µm sections were stained with Bouin's solution for 30 minutes and then stained with Weigert's haematoxylin for 20 minutes and incubated with Biebrich scarlet‐acid fuchsin solution for 20 minutes respectively. The tissue sections were then treated with phosphomolybdic‐phosphotungstic acid solution for 5 minutes, followed by aniline blue solution for 2 minutes. After incubation with 1% acetic acid for 1 minute, the sections were dehydrated with graded alcohol and xylene. Fibrotic areas (blue-stained) within sections were visualized using a light microscope.

**Estimation of total collagen by hydroxyproline assay**

Hydroxyproline assay was performed to measure total collagen content of heart (Stegemann and Stalder, 1967). Briefly, tissue samples were mixed gently with 2N NaOH in a total volume of 50µl. The samples were hydrolyzed by autoclaving at 120°C for 20 mins. Then the samples were cooled to room temperature and 450µl of 0.056M chloramine-T was added to the hydrolysate, mixed gently, and the oxidation was allowed to proceed for 25 mins at room temperature followed by the addition of 1M Ehrlich’s aldehyde reagent, mixed gently, and the chromophore was developed by incubating the samples at 65°C for 20 mins and the absorbance was read at 550nm using a spectrophotometer. With the help of a standard curve, hydroxyproline content in the unknown samples was calculated. The amount of collagen was calculated by multiplying hydroxyproline content by a factor of 8.2.

**Assay of Lipid peroxidation (TBARS)**

Lipid peroxidation (LPO) was determined by the method of Devasagayam and Tarachand (1987). Malondialdehyde (MDA) and other aldehydes have been identified as products of lipid peroxidation, which react with thiobarbituric acid (TBA) to give a pink coloured species that absorbs at 532 nm. The results are expressed as nmol of MDA/mg protein.

**Assay of protein carbonyl content**

Protein carbonyl content in cardiac tissue homogenates were determined by using 2, 4-dinitrophenyl hydrazine (DNPH) with slight modifications of the method of Levine et al. (1990). The absorbance was determined at 366 nm and using the extinction co-efficient of 22 mM^-1^cm^-1^ for aliphatic hydrazones. The results are expressed as nano moles of protein carbonyls/mg protein.

**Assay of SOD and Catalase activity**

The activity of SOD was assayed according to the method of Marklund and Marklund (1974). The degree of inhibition of auto-oxidation of pyrogallol, in an alkaline pH by SOD, was used as a measure of the enzyme activity**.** The enzyme activity is expressed as the amount of enzyme required for 50% inhibition of pyrogallol auto-oxidation/minute/mg protein. The activity of catalase was assayed by the method of Sinha (1972). The method is based on the principle that dichromate in acetic acid is reduced to chromic acetate when heated in the presence of H_2_O_2_ with the formation of perchloric acid as an unstable intermediate. The colour developed was read at 610 nm and the activity of the enzyme is expressed as μmoles of H_2_O_2_ consumed/min/mg protein.

**Assay of Glutathione Peroxidase Activity**

The activity of GPx was determined by the modified method of Rotruck et al. (1973). The remaining GSH after the enzyme catalysed reaction, was complexed with 5, 5' dithiobis-(2-nitrobenzoic acid) (DTNB) that absorbs maximally at 412 nm. 0.4 ml of 0.4M sodium phosphate buffer (pH 7.0), 0.1 ml of 10mM sodium azide, 0.2 ml of 4mM GSH, 0.1 ml of 2.5mM H2O2, 0.1 ml suitably diluted aliquot of the samples and 1.1 ml water were taken into a final incubation volume of 2 ml. The tubes were incubated at 37°C for 3 min and the reaction was terminated by the addition of 0.5 ml of 10% TCA. To determine the remaining reduced glutathione (GSH) content, the supernatant was removed by centrifugation. To 1 ml of supernatant, 3 ml of 0.3M disodium hydrogen phosphate solution and 1 ml of the 0.04% DTNB in 1% sodium citrate were added. The colour developed was read at 412 nm. Suitable aliquots of the standard GSH were taken and treated in the same manner. The enzyme activity is expressed as μmoles of GSH consumed/minute/mg protein.

**Assay of Glutathione Reductase Activity**

Glutathione reductase which utilizes NADPH to convert oxidized glutathione to the reduced glutathione was assayed by the method of Staal et al. (1969). The decrease in the absorbance at 340nm due to the oxidation of nicotinamide adenine dinucleotide phosphate (NADPH) by oxidized glutathione (GSSG) was followed. Glutathione reductase activity was determined by taking 1.5ml of 0.2M sodium phosphate buffer (pH 6.8) and 0.5 ml of 5mM ethylenediamine tetra acetic acid (EDTA) in a final volume of 2.5ml containing 0.2ml of 1mM GSSG, 0.1ml of 120 μM NADPH and suitable aliquot of enzyme. The decrease in absorbance at 340nm at 37oC was measured against the blank for 3 minutes at 1 minute interval. The activity of GR is expressed as μmoles of NADPH consumed/minute/mg protein.

**Determination of GSH/GSSG ratio**

GSH/GSSG ratio were measured using kits purchased from Elabscience Biotechnology Inc., USA. Briefly, the heart tissue samples are weighed, homogenized in the appropriate reagent in the ratio 1:4 (weight: volume) to prepare a 20% homogenate and centrifuged at 10000 g for 10 minutes. GSSG is reduced to GSH by glutathione reductase, and GSH can react with DTNB to produce GSSG and yellow TNB (Ellman, 1959). The amount of total glutathione (GSSG+GSH) determines the amount of yellow TNB. Therefore, the total glutathione was calculated by measuring the OD value at 412 nm and the content of GSSG was determined by first removing GSH from the sample.

**Assay of Glutamate-Cysteine Ligase Catalytic Subunit activity**

Glutamate-Cysteine Ligase Catalytic Subunit (GCLC) was assayed by the method of (Fraser et al., 2003). Reaction mixtures (1.0ml) contained tissue homogenate (200 µl); 100 mM Tris–HCl buffer pH 8.0 (300µl); 150 mM KCl (100µl); 20mM MgCl2 (100 µl); 2 mM EDTA (50 µl); 10 mM ATP (100 µl); 2 mM phosphoenolpyruvate (100 µl), 10 mM L-glutamate (50 µl), 10 mM L-α-aminobutyrate (50 µl), 0.27 mM NADH (50 µl), 2 µg of type II rabbit muscle pyruvate kinase (5 units), and 2 µg of lactate dehydrogenase (10 units). The reaction was initiated by the addition of ATP to a final concentration at 5 mM. The decrease in the absorbance of NADH at 340 nm was monitored for 3 minutes at 30 seconds interval. The enzyme activity was expressed as millimoles of NADH oxidized/min/mg protein.

**Assay of NAD(P)H: Quinone Oxidoreductase 1 activity**

NAD(P)H:Quinone Oxidoreductase1 (NQO1) activity was measured by the method of Merker *et al.* (2006). Briefly, NQO1 activity was calculated by measuring conversion of NADH to NAD^+^ using 2, 6-dichlorophenol indophenol (DCPIP) as substrate. The decrease in NADH was measured at 600 nm for over 1 min. NQO1 activity was measured in 1.0 ml reaction mixture containing 0.5 ml of 25 mM Tris buffer (pH 7.4), 0.1 ml of 200 μM NADH, 0.1 ml of 40 μM DCPIP and 0.2 ml of tissue homogenate. Parallel reactions were performed in the presence of 0.1 ml of 20 μM dicumarol, which inhibits the activity of NQO1. The rate of dicumarol-sensitive NQO1 activity was determined as the difference between the uninhibited and the dicumarol-inhibited rates and was normalized to protein levels. The enzyme activity is expressed as mmoles of DCPIP utilized/min/mg protein.

**Assay of Heme Oxygenase 1 activity**

The enzyme activity of Heme oxygenase-1 (HO-1) was assayed by the method of (Balla *et al*., 1992). The standard incubation mixture in a final volume of 1.0 ml contained 500µl of 10 μmol potassium phosphate buffer (pH 7.4), 100 µl of 60 nmol NADPH, 100 μl microsomal pellet (HO-1 source), 50 μL cytosolic pellet (biliverdin reductase source), and 100 µl of 200 nmol hemin. Incubations were carried out at 37℃ for 60 minutes in dark. After incubation 1.0 ml of chloroform was added to the reaction. The enzyme activity from the reaction mixtures was determined by measuring bilirubin formation, which was calculated as the difference in absorbance measured at 455 and 520 nm, employing a ε value of 50 mM -1cm-1. The concentration of bilirubin produced in 60 minutes was calculated using the extinction coefficient, 40 mmol/L-1cm -1 for bilirubin per mg protein.

**Reverse transcriptase PCR**

Total RNA was isolated from the heart tissue using total RNA isolation reagent (TRIzol reagent) by the method of Chomczynski and Sacchi (1987). Primers were obtained from Sigma–Aldrich (St. Louis, MO, USA). The amplified products were separated by electrophoresis on 2% agarose gel and identified by ethidium bromide staining. Specificity was confirmed by the size of the amplified products with reference to 100 bp DNA ladder (Bio vision, USA) and the band intensities were quantified by Quantity One Software (Bio-Rad, USA). The following set of primers were used in the study.

| **S.No** | **Primer** | **Sequence** | **Product Size** |
| --- | --- | --- | --- |
| **1** | β-MHC | F: CAGTCATGGCGGATCGAGA | 132bp |
|  |  | R: TGTCATCAGGCACAAAAACATCT |  |
| **2** | GCLC | F: TGATTGAAGGGACACCTGGC | 180bp |
|  |  | R: TGTGCTCTGGCAGTGTGAAT |  |
| **3** | NQO1 | F: CGAAGCATTTCAGGGTCGTC | 204bp |
|  |  | R: AGATTCGACCACCTCCCATC |  |
| **4** | HO-1 | F: GAGCGAAACAAGCAGAACCC | 167bp |
|  |  | R: ACCTCGTGGAGACGCTTTAC |  |
| **5** | β-Actin | F: GCCTCTGGTCGTACCACTGGC | 278bp |
|  |  | R: AGGGAGGAAGAGGATGCGGCA |  |

**Western blot analysis**

Samples containing 30-50 μg of proteins were separated by SDS-electrophoresis on 8 –12% polyacrylamide gels and transferred to PVDF (polyvinylidene difluoride) membranes. The membranes were incubated with specific primary antibody against Nrf2, Keap1, Cullin3, GCLC, NQO1, HO-1 and β- actin. After which the membranes were washed with TBS containing 0.05% Tween 20, and incubated with Horseradish Peroxidase (HRP) -conjugated goat anti-rabbit or goat anti-mouse (BioRad, USA) secondary antibody. Immunoreactive bands were developed with Immobilon Western - Chemiluminescent HRP substrate (Millipore Corporation, Billerica, MA, USA) and visualized by using an enhanced chemiluminescence system (Chemi-Doc, BioRad, USA) and presented in comparison to β-actin expression.

**Cell culture**

**Measurement of superoxide radicals**

In the presence of superoxide (O_2_^•−^), the cell-permeable nonfluorescent DHE is oxidized to fluorescent 2-hydroxyethidium, which is then detected by using the excitation/emission filters appropriate for rhodamine. To detect superoxide radical formation, live cells were incubated with 10μM Dihydroethidium (DHE). Nuclei were stained with DAPI (1 μM) for 10 min in the incubator. After incubation, cells were washed with PBS, mounted and images were captured and analysed using Olympus FluoView- 1000 confocal microscope.

**Immunocytochemistry**

Briefly, H9c2 cells were harvested at the end of each experiment at indicated time points, fixed with 3.7% paraformaldehyde in PBS at 4°C for 10 minutes, permeabilized with 0.25% Triton X-100 in TBS-T (0.01%) at room temperature for 10 minutes, and blocked with 5% normal goat serum at room temperature for 30 minutes. For detection of Nrf2, the fixed and permeabilized cells were incubated at 4°C overnight with rabbit anti-Nrf2 primary antibody at a dilution of 1:300 in TBST containing 1% BSA, followed by secondary anti-rabbit Alexa fluor-488-conjugated antibody (1:1,000 dilution) for 1 h at room temperature. The coverslips with cells were then mounted on a glass slide with Fluoromount G mounting medium and viewed with an Olympus FluoView-1000 confocal microscope at × 60 magnification, and data were captured digitally and analysed.

**Cell size measurement**

To measure the cell size, the H9c2 cells on coverslips were stained with the Alexa Fluor 488 phalloidin according to the manufacturer’s guidelines with modification. Briefly, the cells were fixed with 3.7% paraformaldehyde in PBS at 4°C for 10 minutes, permeabilized with 0.25% Triton X-100 in TBS-T (0.01%) at room temperature for 10 minutes, and blocked with 1% BSA at room temperature for 30 minutes followed by the Alexa Fluor 488 phalloidin for 45 minutes at room temperature. Nuclei were counterstained with DAPI. The coverslips were then washed thrice with PBS and mounted on a glass slide with Fluoromount G mounting medium and imaged with an Olympus FluoView-1000 confocal microscope at × 60 magnification, and data were captured digitally and analysed. The cell size was measured using Image J software.

**Supplementary Figure caption and legend**

**Supplementary Figure 1: Cell size measurement.** Representative images of Alexa Fluor 488 Phalloidin staining in H9c2 cells. Nuclei were counterstained with DAPI. Bar plots showing the relative cell size measured using Image J software. Results are shown as the mean ± SEM of three separate experiments. Statistical significance (***: p < 0.001) was calculated by Student-Newman-Keuls and Tukey post hoc tests.

**References**

1. Kiernan JA. 2008. Histological and histochemical methods: theory and practice. 4th ed. Bloxham, UK: Scion
2. Jin L, Lin MQ, Piao ZH, Cho JY, Kim GR, Choi SY, Ryu Y, Sun S, Kee HJ, Jeong MH. Gallic acid attenuates hypertension, cardiac remodeling, and fibrosis in mice with NG-nitro-L-arginine methyl ester-induced hypertension via regulation of histone deacetylase 1 or histone deacetylase 2. Journal of hypertension. 2017 Jul 1;35(7):1502-12.
3. Stegemann H, Stalder K. Determination of hydroxyproline. Clinica chimica acta. 1967 Nov 1;18(2):267-73.
4. Devasagayam TP, Tarachand U. Decreased lipid peroxidation in the rat kidney during gestation. Biochemical and biophysical research communications. 1987 May 29;145(1):134-8.
5. Levine RL, Garland D, Oliver CN, Amici A, Climent I, Lenz AG, Ahn BW, Shaltiel S, Stadtman ER. Determination of carbonyl content in oxidatively modified proteins. Methods in enzymology 1990 Jan 1 (Vol. 186, pp. 464-478).
6. Marklund S,Marklund G. Involvement of the superoxide anion radical in the auto oxidation of pyrogallol and a convenient assay for superoxide dismutase. Eur J Biochem 1974; 47: 469–474.
7. Sinha AK. Colorimetric assay of catalase. Anal Biochem. 1972;47(2):389-394.
8. Rotruck JT, Pope AL, Ganther HE, Swanson AB, Hafeman DG, Hoekstra WG. Selenium: biochemical role as a component of glutathione peroxidase. Science. 1973; 179(4073):588-90.
9. Staal GE, Visser J, Veeger C. Purification and properties of glutathione reductase of human erythrocytes. Biochim. Biophys Acta 1969; 185: 39-48. PubMed PMID: 5796111.
10. Ellman GL. Tissue sulfhydryl groups. Arch Biochem Biophys. 1959;82(1):70-77.
11. Fraser JA, Kansagra P, Kotecki C, Saunders RD, McLellan LI. The modifier subunit of Drosophila glutamate-cysteine ligase regulates catalytic activity by covalent and noncovalent interactions and influences glutathione homeostasis in vivo. J Biol Chem. 2003;278(47):46369-46377.
12. Merker MP, Audi SH, Bongard RD, Lindemer BJ, Krenz GS. Influence of pulmonary arterial endothelial cells on quinone redox status: effect of hyperoxia-induced NAD(P)H:quinone oxidoreductase 1. Am J Physiol Lung Cell Mol Physiol. 2006;290(3):L607-L619.
13. Balla J, Jacob HS, Balla G, Nath K, Vercellotti GM. Endothelial cell heme oxygenase and ferritin induction by heme proteins: a possible mechanism limiting shock damage. Trans Assoc Am Physicians. 1992;105:1-6.
14. Chomczynski P, Sacchi N. Single step method of RNA isolation by acid guanidinium thiocyanate-phenol chloroform extraction. Anal Biochem 1987; 162: 156–159.
