# Supplementary figures and images for: Targeting the Nrf2/ARE Signalling Pathway to Mitigate Isoproterenol-Induced Cardiac Hypertrophy: Plausible Role of Hesperetin in Redox Homeostasis
Source: Oxid Med Cell Longev. 2020 Sep 1;2020:9568278. doi: 10.1155/2020/9568278 (PMC7482027; doi:10.1155/2020/9568278)

Supplementary Figure 1

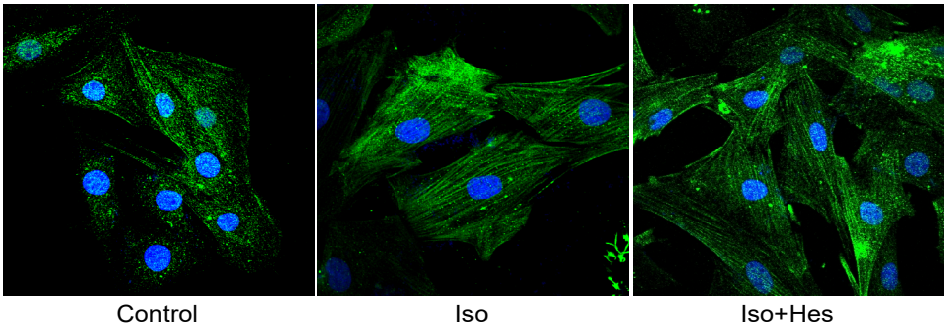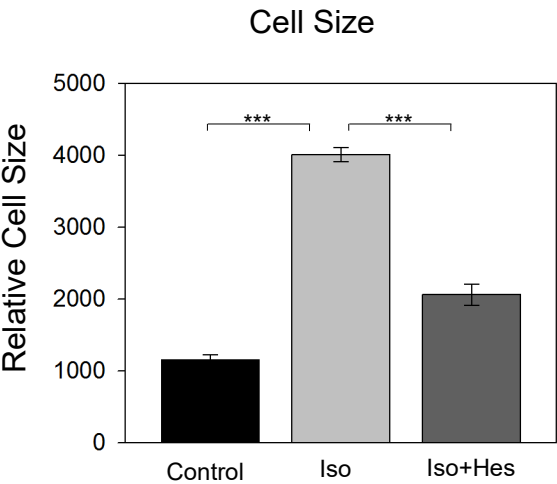

Supplement: Supplementary Materials — Supplementary Figure 1: cell size measurement. Representative images of Alexa Fluor 488 phalloidin staining in H9c2 cells. Nuclei were counterstained with DAPI. Bar plots showing the relative cell size measured using ImageJ software. Results are shown as the mean ± SEM of three separate experiments. Statistical significance (∗∗∗p < 0.001) was calculated by Student-Newman-Keuls and Tukey post hoc tests. [file 9568278.f1.zip › nrf2 supplementary fig1_omcl_3122522.pdf]
